# CD30 expression in extranodal natural killer/T-cell lymphoma, nasal type among 622 cases of mature T-cell and natural killer-cell lymphoma at a single institution in South China

**DOI:** 10.1186/s40880-017-0212-9

**Published:** 2017-05-10

**Authors:** Yanfen Feng, Huilan Rao, Yiyan Lei, Yuhua Huang, Fang Wang, Yu Zhang, Shaoyan Xi, Qiuliang Wu, Jianyong Shao

**Affiliations:** 1 State Key Laboratory of Oncology in South China, Collaborative Innovation Center for Cancer Medicine, Sun Yat-sen University Cancer Center, Guangzhou, Guangdong 510060 P. R. China; 2 Department of Pathology, Sun Yat-sen University Cancer Center, Guangzhou, Guangdong 510060 P. R. China; 3 Department of Thoracic Surgery, The First Affiliated Hospital of Sun Yat-sen University, Guangzhou, Guangdong 510080 P. R. China; 4 Department of Molecular Diagnostics, Sun Yat-sen University Cancer Center, Guangzhou, Guangdong 510060 P. R. China

**Keywords:** Lymphoma, T cell, Natural killer cell, CD30, Epstein-Barr virus

## Abstract

**Background:**

Mature T-cell and natural killer (NK)-cell lymphomas compose a heterogeneous group of non-Hodgkin lymphomas, and extranodal NK/T-cell lymphoma, nasal type (ENKTL) is an aggressive subtype with sporadic CD30 expression. However, the significance of CD30 expression in ENKTL is controversial. We aimed to classify a large cohort of patients with mature T-cell and NK-cell lymphomas according to the 2016 World Health Organization (WHO) classification guidelines and to study the association between CD30 expression and prognosis of patients with ENKTL.

**Methods:**

We selected consecutive patients with mature T-cell and NK-cell lymphomas who attended our institution between September 1, 2009 and August 31, 2013. We classified the lymphomas according to the 2016 revision of the WHO classification of lymphoid neoplasms, analyzed the associations between CD30 expression and clinicopathologic features of ENKTL patients, and evaluated the prognostic implications of CD30 expression.

**Results:**

We identified 622 consecutive patients with mature T-cell and NK-cell lymphomas, including 317 (51.0%) patients with ENKTL. In addition, CD30 expression was detected in 43 (47.3%) of a subset of 91 patients with ENKTL. No clinicopathologic features were associated with CD30 expression, and CD30 positivity showed no prognostic significance in patients with ENKTL.

**Conclusions:**

ENKTL is the most common type of mature T-cell and NK-cell lymphoma diagnosed at our institution. CD30 is frequently expressed in ENKTL and represents a therapeutic target; however, it may not be a prognostic marker.

## Background

Mature T-cell and natural killer (NK)-cell lymphoma is a rare subtype of non-Hodgkin lymphoma. According to the 2008 World Health Organization (WHO) classification of tumors of hematopoietic and lymphoid tissues, the most common subtypes of non-Hodgkin lymphoma are peripheral T-cell lymphoma, not otherwise specified (PTCL-NOS), anaplastic large cell lymphoma (ALCL), angioimmunoblastic T-cell lymphoma (AITL), and extranodal NK/T-cell lymphoma, nasal type (ENKTL) [1]. The subtype incidence and distribution differ according to geographical region and ethnic population. The incidence of mature T-cell and NK-cell lymphoma is relatively high in Asian countries, including China, when compared with those in the United States and Europe [2]. The classification of mature T-cell and NK-cell lymphomas has been modified in the 2016 revision of the WHO classification of lymphoid neoplasms [3].

In general, mature T-cell and NK-cell lymphomas are aggressive tumors with a poor prognosis, and the use of systemic chemotherapy, including anthracycline, achieves complete remission in only a fraction of all cases. The 3-year overall survival (OS) and failure-free survival (FFS) rates of patients with mature T-cell and NK-cell lymphoma are approximately 52% and 32%, respectively [4]. The prognosis of patients with ENKTL is also unsatisfactory, even for those who present with early-stage disease [5]. The recent availability of brentuximab vedotin, an anti-CD30 monoclonal antibody, has led to improved OS and FFS rates among patients with refractory Hodgkin lymphoma, which is characterized by strong and uniform expression of CD30 [6]. A similar benefit was observed for patients with ALCL, a T-cell lymphoma also characterized by the strong and uniform expression of CD30 in neoplastic cells [7]. We previously reported that CD30 is expressed in ENKTL [8]; however, the significance of CD30 expression in ENKTL remains unclear.

Thus, we reviewed clinical records of a large cohort of ENKTL patients and analyzed the prognostic implications of CD30 expression. In addition, we also investigated the subtype distribution and incidence of mature T-cell and NK-cell lymphomas according to the 2016 WHO classification system in a large cancer center in South China.

## Methods

### Patient selection

We searched for all cases of mature T-cell and NK-cell lymphomas diagnosed in the Department of Pathology at the Sun Yat-sen University Cancer Center (Guangzhou, China) between September 1, 2009 and August 31, 2013. Diagnoses were based on the criteria of 2016 revision of WHO Classification of Lymphoid Neoplasms [3]. A series of ENKTL patients who were treated in the Department of Medicine at the Sun Yat-sen University Cancer Center during the same period were retrospectively selected to further investigate their clinical features and CD30 expression levels. The selection criteria were as follows: (1) confirmed diagnosis by pathologists, and (2) availability of complete follow-up records and pathologic materials. Patients with concomitant malignant neoplasms were excluded. Patient records were reviewed, and clinical data regarding age, gender, symptoms at presentation, site of involvement, plasma levels of Epstein-Barr virus (EBV)-DNA determined using quantitative polymerase chain reaction (Q-PCR), clinical stage, response to therapy, and status at last follow-up were extracted. The Institutional Review Board at the Sun Yat-sen University Cancer Center approved this study.

### Patient follow-up

All patients were followed up every 6 months until May 2015. Disease progression and recurrence were diagnosed based on clinical examination, imaging assessments, and pathologic examination. OS was measured from the initiation of treatment to either the last follow-up or death from any cause. Progression-free survival (PFS) was measured from the initiation of treatment to the first indications of disease progression or relapse, death from any cause, or the last follow-up.

### Histopathologic and immunohistochemical analysis

All the diagnoses were confirmed by seven senior pathologists from the Department of Pathology at the Sun Yat-sen University Cancer Center after reviewing hematoxylin and eosin-stained tissue sections, immunohistochemistry (IHC) results, and other ancillary materials (including clinical records). A diagnostic consensus was made when the preliminary review by individual pathologists reached inconsistent diagnoses. IHC was performed on sections of 4-μm thickness. To determine the immunophenotype, IHC examination and in situ hybridization for Epstein-Barr virus-encoded small RNAs (EBERs) were performed. The main primary antibodies and antigen retrieval methods for each antibody are shown in Table 1. Appropriate positive and negative controls were included for each antibody.

**Table 1:** Primary antibodies and conditions used for immunohistochemical staining

Primary antibody	Manufacturer (Cat. No.)	Pretreatment	Working dilution
Mouse anti-CD20 monoclonal antibody	Zymed, San Diego, CA, USA (18-0155)	HP CB (pH 6.0)	1:100
Mouse anti-CD79A monoclonal antibody	Novocastra, New Castle, Tyne and Wear, UK (RTU-CD79α-192)	HP CB (pH 6.0)	Ready to use
Mouse anti-CD3ε monoclonal antibody	Novocastra, New Castle, Tyne and Wear, UK (RTU-CD3-PS1)	HP CB (pH 6.0)	Ready to use
Mouse anti-CD45RO monoclonal antibody	Zymed, San Diego, CA, USA (08-1365)	HP CB (pH 6.0)	Ready to use
Mouse anti-CD5 monoclonal antibody	Zymed, San Diego, CA, USA (08-1283)	HP CB (pH 6.0)	Ready to use
Mouse anti-CD10 monoclonal antibody	Novocastra, New Castle, Tyne and Wear, UK (RTU-CD10-270)	HP CB (pH 6.0)	Ready to use
Mouse anti-CD23 monoclonal antibody	Novocastra, New Castle, Tyne and Wear, UK (RTU-CD23-1B12)	HP CB (pH 6.0)	Ready to use
Mouse anti-CD30 monoclonal antibody	Zymed, San Diego, CA, USA (08-0155)	HP CB (pH 6.0)	Ready to use
Mouse anti-CD56 monoclonal antibody	Zymed, San Diego, CA, USA (08-0152)	HP CB (pH 6.0)	Ready to use
Mouse anti-Bcl-6 monoclonal antibody	Zymed, San Diego, CA, USA (08-1426)	HP EDTA (pH 9.0)	Ready to use
Mouse anti-ALK monoclonal antibody	Zeta, Sierra Madre, CA, USA (Z2035)	HP EDTA (pH 8.0)	Ready to use
Mouse anti-Ki-67 monoclonal antibody	Zymed, San Diego, CA, USA (18-0192Z)	HP CB (pH 6.0)	1:50
Mouse anti-TdT monoclonal antibody	Zymed, San Diego, CA, USA (18-7237)	HP EDTA (pH 9.0)	1:50
Mouse anti-TIA-1 monoclonal antibody	Zeta, Sierra Madre, CA, USA (Z2183)	HP CB (pH 6.0)	Ready to use
Mouse anti-Perforin monoclonal antibody	Zeta, Sierra Madre, CA, USA (Z2118)	HP CB (pH 6.0)	Ready to use
Mouse anti-Granzyme B monoclonal antibody	Newmarket Scientific, Newmarket, Suffolk, UK (MS-1157-s)	HP CB (pH 6.0)	Ready to use
Mouse anti-PD-1 monoclonal antibody	Zymed, San Diego, CA, USA (0381)	HP CB (pH 6.0)	1:100
Goat anti-CXCL13 polyclonal antibody	Zymed, San Diego, CA, USA (0043)	HP CB (pH 6.0)	1:100
Mouse anti-CD4 monoclonal antibody	Zymed, San Diego, CA, USA (0032)	HP CB (pH 6.0)	1:200
Rabbit anti-CD8 monoclonal antibody	Zymed, San Diego, CA, USA (0043)	HP CB (pH 6.0)	1:100

*HP* high pressure (boiled with buffer in a stainless steel high-pressure cooker for 10 min, with the pressure setting at approximately 103 kPa), *CB* 0.01 mol/L citrate buffer, *EDTA* 0.01 mol/L ethylenediaminetetraacetic acid, *Bcl-6* B-cell lymphoma-6, *ALK* anaplastic large cell lymphoma kinase, *TdT* terminal deoxynucleotidyl transferase, *TIA-1* T-cell-restricted intracellular antigen-1, *PD-1* programmed cell death 1, *CXCL13* C-X-C motif chemokine ligand 13

The EBV Probe In Situ Hybridization Kit (DIG-AP, A300K.9901, PanPath Company, Amsterdam, the Netherlands) was used to detect EBERs according to the following procedure: (1) deparaffinization and rehydration of the paraffin sections using xylene and a series of graded ethanol solutions, respectively; (2) pretreatment of samples with 0.4% pepsin for 10 min; (3) hybridization with digoxigenin-conjugated EBV probes at 37 °C for 3 h; (4) signal detection using peroxidase-conjugated anti-digoxigenin antibody and 3,3′-diaminobenzidine (DAB); and (5) counterstaining of sections with a hematoxylin solution. The positive signals were brownish-yellow in color and localized within the nuclei.

CD30 was detected using a ready-to-use antibody supplied by Zymed Laboratories (Zymed-08-155, Thermo Fisher Scientific Company, South San Francisco, CA, USA). IHC was performed on sections of 4-μm thickness. Heat-induced antigen retrieval was performed using a citrate acid buffer (pH 6.0) in a stainless steel high-pressure cooker for 10 min, with the pressure set to approximately 103 kPa. The antibody was detected using DAB on a DAKO EnVision system (Dako, Carpinteria, CA, USA) with hematoxylin as a counterstain. CD30 expression was semi-quantitated in positive tumor cells. A total of 100 tumor cells in two randomly selected high-power fields were counted, and the average percentage of positive cells was calculated as the rate of CD30 expression. CD30 positivity was defined as strong cell membrane and Golgi zone reactivity in ≥20% of tumor cells [9].

### Statistical analysis

Categorical variables were compared using either the Chi square or Fisher’s exact tests. OS and PFS were estimated using the Kaplan–Meier method. Differences were considered significant when the two-sided *P* value was less than 0.05. All analyses were performed with SPSS 19.0 software (SPSS, Inc. Chicago, IL, USA).

## Results

### Subtype distribution

**Fig. 1:**
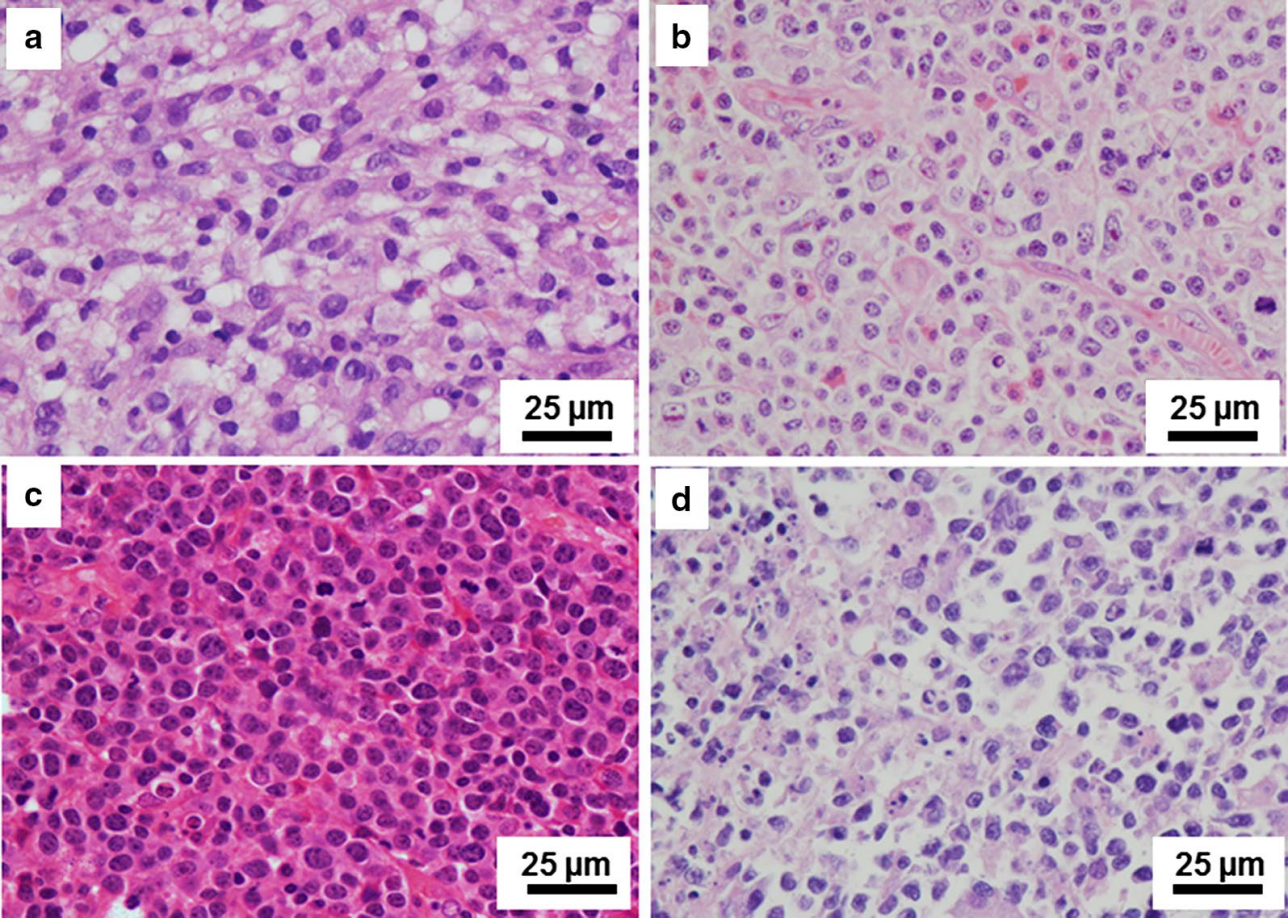
Morphology of different subtypes of T-cell and natural killer (NK)-cell lymphoma (hematoxylin–eosin staining, ×400). **a** Extranodal NK/T cell lymphoma, nasal type (ENKTL) shows medium-sized tumor cells with apoptotic bodies. **b** Angioimmunoblastic T-cell lymphoma (AITL) shows proliferation of high endothelial venules, polymorphic infiltrating cells, and cells with clear-to-pale cytoplasm. **c** Peripheral T-cell lymphoma, not otherwise specified (PTCL-NOS) shows medium- to large-sized polymorphic tumor cells with high mitotic figure rates. **d** Anaplastic lymphoma kinase (ALK)-positive, anaplastic large-cell lymphoma (ALCL) shows cells with horseshoe or kidney-shaped nuclei and inflammation

In total, 622 patients with mature T-cell and NK-cell lymphomas were identified during the study period. The median patient age was 47.5 years (range 2–87 years); 420 were males, and 202 were females (male-to-female ratio = 2.1:1). The distribution of common histological subtypes was as follows: 317 (51.0%) patients had ENKTL (Fig. 1a), 118 (19.0%) had AITL (Fig. 1b), 81 (13.0%) had PTCL-NOS (Fig. 1c), 31 (5.0%) had anaplastic lymphoma kinase (ALK)-positive ALCL (Fig. 1d), 15 (2.4%) had ALK-negative ALCL, and 13 (2.1%) had primary cutaneous ALCL (Table 2).

**Table 2:** Immunophenotype distribution in 622 cases of mature T-cell and NK-cell lymphoma

Immunophenotype	Total [cases (%)]	Male/female ratio	Age [years, median (range)]
Extranodal NK/T-cell lymphoma, nasal type	317 (51.0)	217/100	43.0 (10–86)
Angioimmunoblastic T-cell lymphoma	118 (19.0)	82/36	63.0 (36–87)
Peripheral T-cell lymphoma, not otherwise specified	81 (13.0)	58/23	49.0 (25–81)
Anaplastic large-cell lymphoma, ALK-positive	31 (5.0)	19/12	21.0 (4–69)
Anaplastic large-cell lymphoma, ALK-negative	15 (2.4)	10/5	45.0 (8–76)
Primary cutaneous, anaplastic large-cell lymphoma			48.0 (24–81)
	13 (2.1)	4/9	7.5 (2–13)
Hydroa vacciniforme-like LPD	12 (1.9)	8/4	
Subcutaneous panniculitis-like T-cell lymphoma	12 (1.9)	5/7	36.0 (17–55)
Monomorphic epitheliotropic intestinal T-cell lymphoma	9 (1.4)	6/3	50.0 (38–82)
Hepatosplenic T-cell lymphoma	5 (0.8)	4/1	37.0 (29–44)
Primary cutaneous CD4-positive small/medium T-cell LPD	3 (0.4)	3/0	40.0 (22–60)
Primary cutaneous γδ T-cell lymphoma	2 (0.3)	1/1	19.0 (2–36)
Mycosis fungoides	2 (0.3)	1/1	29.0 (18–40)
Aggressive NK-cell leukemia	2 (0.3)	2/0	27.0 (22–32)
Total	622	420/202	47.5 (2–87)

*NK* natural killer, *ALK* anaplastic lymphoma kinase, *LPD* lymphoproliferative disorder

The most common tumor site among all the patients was the upper aerodigestive tract (*n* = 263, 42.3%), followed by the lymph nodes (*n* = 189, 30.4%), skin (*n* = 70, 11.3%), and gastrointestinal tract (*n* = 37, 5.9%). Other less common sites are listed in Table 3.

**Table 3:** Primary sites of 622 cases of mature T-cell and NK-cell lymphoma

Primary site	Case(s)	Percentage (%)
Upper aerodigestive tract	263	42.3
Lymph nodes	189	30.4
Skin	70	11.3
Gastrointestinal tract	37	5.9
Soft tissue	11	1.8
Bone marrow	10	1.6
Orbit	7	1.1
Omentum	6	1.0
Spleen	6	1.0
Chest	3	0.5
Lung	3	0.5
Testicle	3	0.5
Bone	2	0.3
Liver	2	0.3
Ovary	2	0.3
Parotic gland	2	0.3
Prostate	2	0.3
Brain	1	0.2
Breast	1	0.2
Pancreas	1	0.2
Adrenal gland	1	0.2
Total	622	100

Among the 317 cases of ENKTL, 240 (75.7%) located in the upper aerodigestive tract [164 (51.6%) in the nasal cavity, 39 (12.3%) in the nasopharynx, 20 (6.3%) in the oropharynx, 10 (3.4%) in the oral cavity, and 7 (2.2%) in the throat], 20 (6.3%) in the gastrointestinal tract, and 13 (4.1%) in the skin. Less commonly involved sites included the soft tissue, orbit, pancreas, ovary, prostate, and testis. Only 5 patients with ENKTL had primary nodal involvement; nasal cavity or nasopharyngeal involvement was later discovered in 4 of these 5 patients. Among the 118 patients with AITL, 110 (92.4%) had nodal involvement, whereas among the 81 patients with PTCL-NOS, 39 (47.6%) had nodal involvement.

In total, 91 patients with ENKTL met the selection criteria mentioned above, and their data were collected along with available paraffin blocks and unstained slides to study the association between clinicopathologic features and CD30 expression.

### Clinical features of ENKTL

The clinical features of 91 patients with ENKTL are shown in Table 4. The upper aerodigestive tract, in particular the nasal cavity, was the most common site of presentation, followed by the skin, orbit, and gastrointestinal tract. The distribution of clinical stages was as follows: stage I disease in 37 (40.7%) patients, stage II in 34 (37.4%) patients, stage III in 5 (5.5%) patients, and stage IV in 15 (16.4%) patients. Forty-one (45.1%) patients had regional lymph node involvement. Eighty-one patients were tested for plasma EBV DNA using Q-PCR before treatment: 39 (48.1%) were positive, with EBV DNA loads ranging from 1.07 × 10^3^ to 2.49 × 10^6^ copies/mL; 42 (51.9%) were negative for viral loads.

**Table 4:** Clinical features of 91 patients with extranodal natural killer (NK)/T-cell lymphoma, nasal type

Item	Case(s)	Percentage (%)
Sex		
Female	23	25.3
Male	68	74.7
Age (years)		
≤60	81	89.0
>60	10	11.0
B symptoms		
No	46	50.5
Yes	42	46.2
Unavailable	3	3.3
Primary sites		
Upper aerodigestive tract	78	85.7
Skin	5	5.5
Orbit	3	3.3
Gastrointestinal tract	2	2.2
Lymph node	1	1.1
Breast	1	1.1
Lung	1	1.1
Ann Arbor stage		
Stage I	37	40.7
Stage II	34	37.4
Stage III	5	5.5
Stage IV	15	16.4
Lymph node involvement		
Yes	41	45.1
No	50	54.9

### Microscopic and immunohistochemical features of ENKTL

Microscopically, ENKTL presented with necrosis, ulcerations, angiocentricity, and inflammation. The tumor cells were small, medium, or large in size and displayed irregular nuclear contours and granular chromatin.

**Fig. 2:**
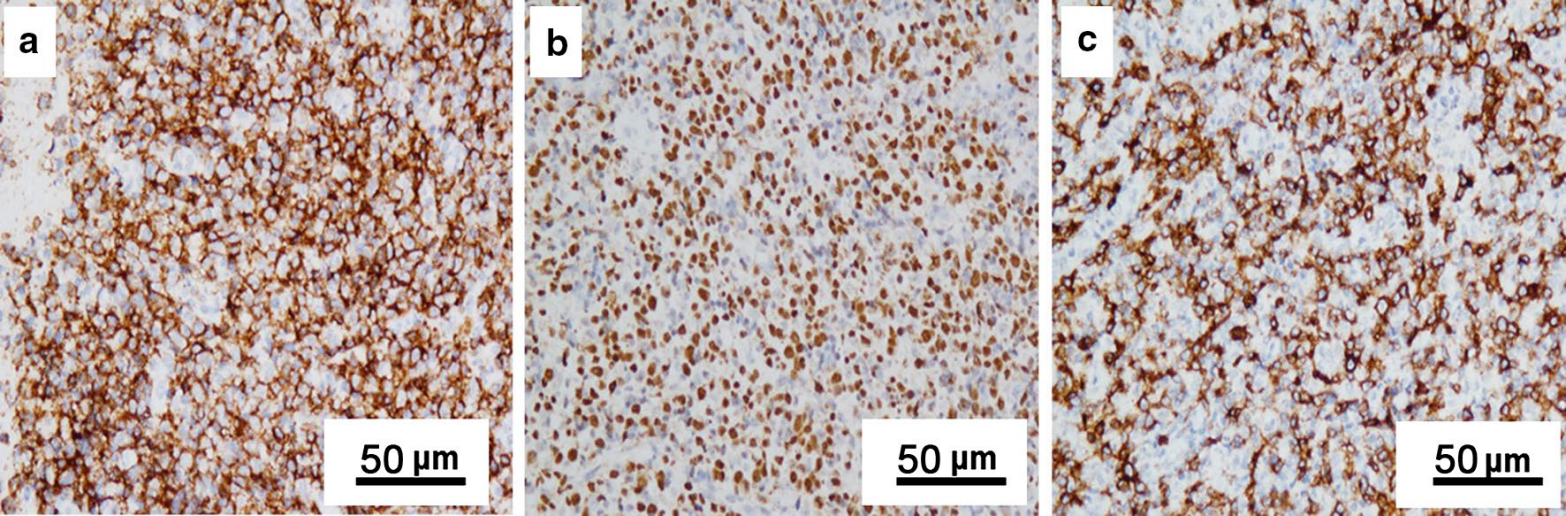
Immunohistochemical staining (IHC) and in situ hybridization (ISH) staining of ENKTL. **a** Tumor cells show strong membranous and cytoplasmic expression of CD56 (IHC, ×200). **b** Tumor cells show strong nuclear expression of Epstein-Barr virus (EBV)-encoded small RNA (ISH, ×200). **c** Tumor cells show strong diffuse membranous and Golgi zone expression of CD30 (IHC, ×200)

Immunohistochemistry studies revealed that 85 (93.4%) of the 91 cases stained positive for the NK-cell marker CD56 (Fig. 2a). All cases were positive for cytoplasmic marker CD3ε and cytotoxic markers T-cell intracellular antigen-1 (TIA-1), granzyme B, and Perforin. CD5 expression was positive in 17 (20.2%) of 84 cases. CD4 and CD8 expression was investigated in 45 cases: 40 (88.9%) were CD4-negative and CD8-negative, 4 (8.8%) were CD4-negative and CD8-positive, and 1 (2.2%) was CD4-positive and CD8-negative. EBER was strongly and diffusely detected in the nuclei of neoplastic cells among all cases (Fig. 2b). CD30 expression was observed in 65 (71.4%) cases. In 43 (47.3%) cases, CD30 was detected in ≥20% of cells; based on the criteria, these cases were determined to be CD30-positive (Fig. 2c).

Fifty patients underwent bone marrow examination, and one had bone marrow involvement.

### Treatment and survival

Of the 91 patients with ENKTL, 58 (63.7%) were treated with chemoradiotherapy, 29 (31.7%) with chemotherapy alone, 2 (2.2%) with radiotherapy alone, 1 (1.1%) with chemotherapy plus surgery (right hemicolectomy); the treatment modality was unknown for 1 patient. The most commonly used chemotherapy regimen was cyclophosphamide, doxorubicin, vincristine, and prednisone (CHOP). Thirty-one patients were treated with either pegaspargase or l-pegaspargase. Of the 71 patients with early-stage (stage I/II) disease, 56 were treated with chemoradiotherapy, 13 with chemotherapy alone, and 2 with radiotherapy alone. Of the 20 patients with advanced (stage III/IV) disease, 16 were treated with chemotherapy alone, 2 with chemoradiotherapy, and 1 with chemotherapy plus surgery; the treatment modality was unknown for 1 patient. For patients who underwent radiotherapy (either radiotherapy alone or in combination with chemotherapy), the median dose was 55 Gy (range 40–66 Gy).

**Fig. 3:**
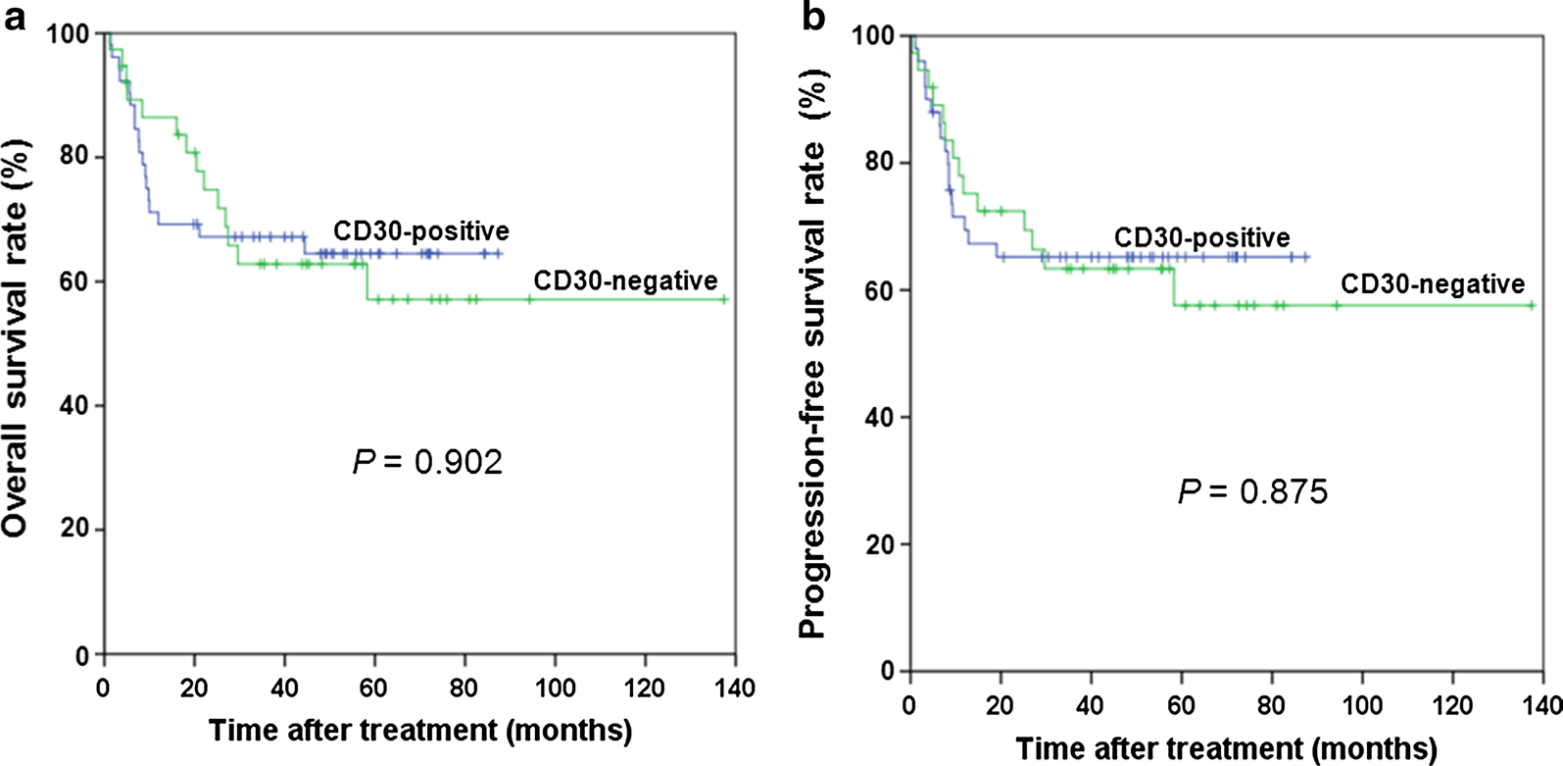
Survival curves of 91 patients with ENKTL as stratified by CD30 expression. **a** Overall survival; **b** Progression-free survival

The median follow-up time was 38.5 months (range 1.1–137.1 months). The median OS was 37.8 months [95% confidence interval (CI), 26.0–49.3 months]. The 5-year OS rate was 66.0%, with 28 (30.8%) patients died during follow-up. The median PFS was 20.8 months (95% CI, 16.5–31.2 months), and the 5-year PFS rate was 63.0%. There was no association between CD30 positivity and either OS or PFS (both *P* > 0.05) (Fig. 3).

**Fig. 4:**
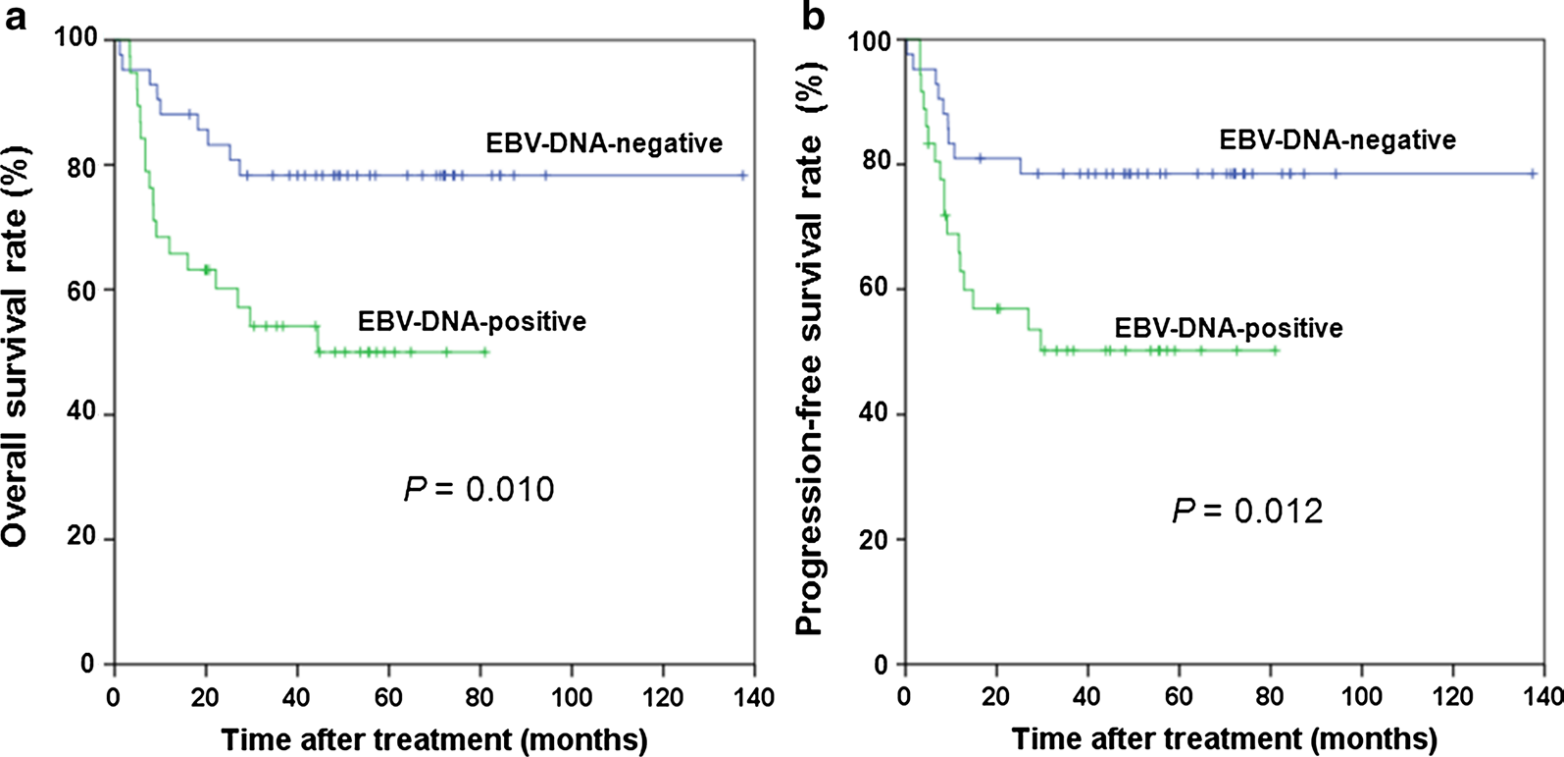
Survival curves of 81 patients with ENKTL as stratified by plasma EBV-DNA status. **a** Overall survival; **b** Progression-free survival

There was also no association between CD30 positivity and age, sex, B symptoms, Ki-67 index, clinical stage, Ann-Arbor stage, lactate dehydrogenase level, primary site, therapeutic modality, plasma EBV-DNA status, or relapse (Table 5). However, patients who tested positive for plasma EBV-DNA had longer OS (*P* = 0.008) and PFS (*P* = 0.012) than patients negative for plasma EBV DNA (Fig. 4).

**Table 5:** Association between CD30 expression and clinicopathologic features of patients with extranodal natural killer/T-cell lymphoma, nasal type

Variable	CD30 expression [cases (%)]		*P* value
	Positive	Negative	
Total	43	48	
Age (years)			0.508
≤60	37 (40.6)	44 (48.4)	
>60	6 (6.6)	4 (4.4)	
Sex			0.675
Male	33 (36.3)	35 (38.5)	
Female	10 (10.9)	13 (14.3)	
B symptoms			0.627
Yes	21 (23.1)	21 (23.1)	
No	22 (24.1)	27 (29.7)	
Ki-67 index			0.060
≤50	14 (15.4)	25 (27.5)	
>50	29 (31.8)	23 (25.3)	
Primary site			0.720
Upper aerodigestive tract	37 (40.6)	40 (44.0)	
Others	6 (6.6)	8 (8.8)	
Ann-Arbor stage			0.080
I/II	37 (40.6)	34 (37.4)	
III/IV	6 (6.6)	14 (15.4)	
LDH level			0.894
Elevated	12 (13.2)	14 (15.4)	
Normal	31 (34.0)	34 (37.4)	
Therapeutic modality			0.230
Chemotherapy alone	10 (10.9)	19 (20.9)	
Chemoradiotherapy	31 (34.0)	27 (29.7)	
Radiotherapy alone	1 (1.1)	1 (1.1)	
Chemotherapy plus surgery	1 (1.1)	0 (0.0)	
Unavailable	0 (0.0)	1 (1.1)	
EBV-DNA status			0.602
Positive	19 (20.9)	20 (22.0)	
Negative	18 (19.8)	24 (26.3)	
Unavailable	6 (6.6)	4 (4.4)	
Relapse			0.657
Yes	19 (20.9)	19 (20.9)	
No	24 (26.3)	29 (31.9)	

*LDH* lactate dehydrogenase, *EBV* Epstein-Barr virus

## Discussion

Here we report a large cohort of 622 patients with mature T-cell and NK-cell lymphomas from a single institution in South China and outlined a basic subtype distribution according to the revised 2016 WHO classification system. We identified ENKTL as the most common mature T-cell and NK-cell lymphoma subtype (51.0%) and confirmed a high frequency of CD30 expression (47.3%) in ENKTL cases.

According to the 2008 WHO classification of tumors of hematopoietic and lymphoid tissues, mature T-cell and NK-cell lymphomas compose a group of heterogeneous lymphomas, including ENKTL, AITL, ALCL, PTCL-NOS, and other rare subtypes [1]. Recently, a revision of the WHO classification of lymphoid neoplasms and the accompanying monograph has been published [3]. This revised edition included refinements of rare types of lymphoma and introduced molecular changes in the definition of certain subtypes. Herein we classified 622 cases of mature T-cell and NK-cell lymphoma according to this revised WHO classification, which yielded new incidence data based on a large institutional study.

Within this cohort, ENKTL was the most common subtype, accounting for 51.0% of cases over a 4-year period. Similar frequencies have been reported in North China [10] and Japan [11], but these frequencies are much higher than those reported in the United States and in European countries [2]. Recently, Li et al. [12] identified the genetic risk of ENKTL based on a genome-wide association study of cases and controls from Guangdong province in South China, which indicated a genetic predisposition to ENKTL among this population.

CD30 is a member of tumor necrosis factor (TNF) receptor super-family and exerts numerous biological functions, including cell cycle arrest, apoptosis, and activation of the pro-survival transcription factor nuclear factor kappa-light-chain-enhancer of activated B cells (NF-κB). CD30 is preferentially expressed in activated B cells but is also expressed in T cells and NK cells [13]. Among malignant lymphomas, CD30 is expressed in Hodgkin and Reed-Sternberg cells of Hodgkin lymphoma and in almost all neoplastic cells of ALCL. Several studies with small case series have reported CD30 expression in mature T-cell and NK-cell lymphoma, with the reported percentage of ENKTL patients that were CD30-positive ranging from 36.4% to 41% [10, 14–16]. In the current study, CD30 was expressed in 47.3% of cases, which is much higher than the rate reported in another study of mature T-cell and NK-cell lymphomas (with the exception of ALCL) [17]. CD30 has been reported to be more frequently expressed in ENKTL than in other subtypes of mature T-cell and NK-cell lymphomas, and this may be due to the concomitant presence of EBV in ENKTL patients [1, 18]. A type II latency pattern of EBV infection has been observed in ENKTL patients, whereby tumor cells express EBERs, EBV nuclear antigen-1 (EBNA-1), latent membrane protein (LMP)-1, and LMP-2. LMP-1 is the most critical EBV element involved in cell proliferation. It induces the expression of B-cell lymphoma-2 (Bcl-2) and TNF-α-induced protein-3 to inhibit apoptosis. LMP-1 also up-regulates c-Myc, interleukin (IL)-6, and IL-10 to accelerate cell proliferation. LMP-1 has also been shown to up-regulate CD30 [18]; thus, LMP-1 expression may be associated with the high rate of CD30 expression among patients with ENKTL.

The prognostic significance of CD30 expression in lymphomas is not well reported. Hu et al. [9] reported that CD30 expression was associated with a favorable prognosis among patients with EBV-positive diffuse large B-cell lymphoma. Bisig et al. [19] reported that patients with CD30-positive PTCL-NOS tended to have a better clinical outcome than those with CD30-negative PTCL-NOS. However, the prognostic value of CD30 expression among patients with ENKTL is controversial. In one study comprising 36 patients with ENKTL, CD30 expression appeared to be associated with a favorable outcome [10]. In another study of 22 patients with ENKTL, Hong et al. [16] reported that CD30 expression was associated with a poor prognosis. Kim et al. [14] (*n* = 72) and Kuo et al. [15] (*n* = 22) revealed that there was no difference in OS among CD30-positive patients versus CD30-negative patients. In our study, CD30 expression did not exhibit a prognostic significance. Since we included more cases than most (if not all) studies in the current literature, we speculate that CD30 may not be a prognostic marker for ENKTL. It is likely that a selection bias exists in our cohort because patients in our study tended to present at earlier disease stages than patients in other studies. Studies with larger referral bases are needed to determine the significance of CD30 on patient prognosis.

Although it does not appear that CD30 expression has a prognostic role in ENKTL, CD30 is a molecular target for brentuximab vedotin, an anti-CD30 monoclonal antibody, which has led to improved outcomes in patients with refractory classic Hodgkin lymphoma and ALCL [6, 20, 21]. Horwitz et al. [22] observed objective responses to brentuximab vedotin in patients with relapsed T-cell lymphomas, and these responses were seen among patients regardless of their CD30 expression status in the tumor samples. More recent researches also showed that treatment with brentuximab vedotin led to complete remission in patients with CD30-positive refractory extranodal NK/T-cell lymphoma [23] and cutaneous T-cell lymphoma [24]. Therefore, targeted therapy with an anti-CD30 antibody represents a promising management of patients with refractory ENKTL that expresses CD30. A phase 2 clinical trial is currently in processing for patients with T-cell lymphomas (including ENKTL) to determine the effect of brentuximab vedotin on CD30-positive cases [25].

Other potential therapeutic modalities include targeting EBV antigens or using adoptive immune therapy in patients with circulating EBV DNA [26]. In our study cohort, plasma EBV DNA was detected in 48.1% of the tested cases prior to treatment initiation; the presence of EBV DNA was associated with a poorer prognosis. This result is similar to that of a previous report [27]. Plasma EBV DNA is derived from apoptotic tumor cells. It is an accurate surrogate biomarker of lymphoma load and a prognostic factor for patients treated with conventional chemotherapy [28]. However, we did not observe an association between plasma EBV DNA load and CD30 expression.

In conclusion, ENKTL is the most common subtype of mature T-cell and NK-cell lymphoma treated at our institution, a reference cancer center in South China. We identified that CD30 was expressed in a high percentage of ENKTL cases and might be a potential therapeutic molecular target for this disease; however, CD30 was insufficient as a prognostic marker. Clinical trials are needed to determine the effect of anti-CD30 therapy in patients with ENKTL that express CD30.
